# Trends in drug offers among adolescents in the United States, 2002–2014

**DOI:** 10.1186/s40352-017-0051-4

**Published:** 2017-05-30

**Authors:** Sehun Oh, Christopher P. Salas-Wright, Michael G. Vaughn

**Affiliations:** 1 School of Social Work, The University of Texas at Austin, Austin, TX USA; 2 School of Social Work, Boston University, Boston, MA USA; 3 School of Social Work, College for Public Health and Social Justice, Saint Louis University, St. Louis, MO USA

**Keywords:** Drug offer, Adolescence, Trends, African-American, Hispanic

## Abstract

**Background:**

Being offered illicit drugs is a critical factor leading to drug initiation and other psychosocial risk behaviors among adolescents in the United States. However, there exist few studies examining the recent trends in drug offers among adolescents, particularly across racial/ethnic subgroups. The present study examines trends and psychosocial/behavioral correlates of drug offers among adolescents of the three largest racial/ethnic groups.

**Methods:**

We used data from the 2002–2014 National Survey on Drug Use and Health of adolescents aged 12–17, which include African-American, Hispanic, and White adolescents (*n* = 199,700) in the U.S. We estimated the prevalence of past-month drug offers by race/ethnicity, and conducted logistic regression analyses to test the significance of the trends and to examine the correlates of drug offers.

**Results:**

Overall, the prevalence of drug offers decreased significantly from 16.3% in 2002 to 12.3% in 2014, reflecting a 24.5% reduction in the relative proportion of adolescents who were offered drugs. While the decreasing trends were observed in all subgroups (e.g., race/ethnicity), the decreases were more limited among African-American and Hispanic youth than White youth. As a result, while no differences were observed at the outset of the study, a higher proportion of African-American and Hispanic adolescents were offered drugs between 2012 and 2014.

**Conclusions:**

Findings suggest a general decline in drug offers among adolescents in the U.S., but racial/ethnic differences in prevalence were identified. This underscores the importance of further efforts to understand the racial/ethnic differences in drug offers and suggests the need for culturally-sensitive drug prevention programs.

## Background

Adolescent drug use is known as one of the most detrimental risk behaviors threatening the current and future well-being of youths. Alcohol and drug use is not only considered as one of the leading causes of mortality, but also a key contributors of suicide, homicide, poisoning, and the spread of infectious disease among youths around the world (Salas-Wright et al. 2017b). Despite the deleterious effects on well-being of youths, a disconcerting proportion of youths in the United States continue to use drugs (Johnson et al. 2015; Salas-Wright et al. 2015). For instance, 14% of 10th graders and 22% of 12th grades in the U.S. reported past-month marijuana use in 2016 (National Institute on Drug Abuse 2017). Moreover, only a minor decline or no significant changes in illicit drug use were found in the past decade, in contrast to continuous decreases in alcohol and cigarette use (National Institute on Drug Abuse 2017; Salas-Wright et al. 2015). When examined separately by race/ethnicity, the differences among African-Americans, Hispanics, and Whites have narrowed as more African-Americans are now using marijuana while the proportion remains relatively stable among Whites. (Johnston et al. 2017). The differential trend patterns across racial/ethnic subgroups suggest a racially and ethnically sensitive examination of adolescent substance use and related factors (Chen & Jacobson 2012; Shih et al. 2010).

As a part of efforts to understand why adolescents initiate drug use, previous research has uncovered a number of predictors, such as other substance use (e.g., alcohol and tobacco; Caris et al. 2009), family factors (e.g., parental monitoring, family relationship quality; Van Ryzin et al. 2012), school disengagement (Henry et al. 2012), peer pressure and deviant peer association (Andreas & Pape 2015; Pederson et al. 2013), and drug offers (Ellickson et al. 2004; Manning et al. 2001; Siegel et al. 2015). Of these factors, drug offers are considered to have substantial impact on adolescent drug use as drugs offers are considered as “the first step of involvement in drug use” (Benjet et al. 2007, p. 128) and it magnifies drug use cues (Wertz & Sayette 2001). For instance, Manning et al. (2001) found that 65.9% of adolescent marijuana users in South London reported drug offer as the major reason for their initiation of marijuana use. Similarly, Siegel et al. (2015) also found that being offered marijuana was a significant predictor of current and 1-year later marijuana initiation among youth.

In an attempt to understand why some adolescents are more likely to receive illicit drug offers, prior studies, mostly based on non-U.S. samples, identified a number of risk factors and correlates of drug offers. Identified factors primarily represent different aspects of delinquency, which are also considered as a signal for other antisocial behaviors, and interpersonal relations (Andreas & Pape 2015). The delinquency-related risk factors include underage use of alcohol and tobacco (Caris et al. 2009; Wagner & Anthony 2002), theft (Andrea & Pape 2015), and aggressiveness (Rosenberg & Anthony 2001), supported by the externalizing spectrum of behavior that posits adolescents of higher externalizing behaviors are more likely to be exposed to illicit drugs (Krueger et al. 2002; Vaughn et al. 2014). For interpersonal relations, parental factors (i.e., parental warmth/control and conflict) and school relations (e.g., drug-using peers) were two primary factors leading to higher drug offers (Neumark et al. 2012; Prado et al. 2009). Although few prior studies examined the direct association between academic factors and drug offers, the relationship between drug use and school readiness (often measured by basic academic skills such as reading or math scores) supports the possible link between academic factors and drug offers (Storr et al. 2011). Moreover, religiosity is expected to act as a protective factor of drug offers given its positive impacts on self-control as well as buffering effects between risk behaviors and substance use (Salas-Wright et al. 2016, 2017a).

Despite substantial evidence regarding the impact of drug offers on adolescent substance use and associated risk factors, little research has accrued examining how drug offers may or may not have changed in recent years. In addition, there exist few recent U.S.-based studies (e.g., Storr et al. 2011) that examined psychosocial and behavioral correlates of drug offers. Understanding the trends of drug offers and its association with psychosocial and behavioral risks is critical in informing strategies to address adolescent drug use and to reduce associated risks. Thus, the aim of present study is to examine recent trends and psychosocial and behavioral correlates of drug offers among adolescents of the three largest racial/ethnic groups in the U.S. (i.e., African-American, Hispanic and White youth). Specifically, this study intends to answer to the following research questions: (1) Have drug offers among adolescents in the U.S. significantly changed since 2005? (2) Have any racial/ethnic disparities existed in the trends of drug offers during the same time period? (3) What key psychosocial and behavioral risks are correlated with drug offers?

## Methods

### Data and procedures

This study uses data from the National Survey on Drug Use and Health (NSDUH) between 2002 and 2014. The NSDUH is administered by the Substance Abuse and Mental Health Services Administration (SAMHSA) and provides population estimates for substance use and a wide range of health behaviors among the U.S. civilian, noninstitutionalized population aged 12 and older. The sample was selected using multistage stratified sampling design where the sample was divided into eight “large” states and 43 “small” states to yield 3600 and 900 respondents per state, respectively (SAMHSA 2014). The 2005–2014 surveys were conducted using computer-assisted interviewing methodology and identical measures across all survey years.

To examine the trend in the prevalence and health-related correlates among adolescents of major racial/ethnic groups in the U.S., 199,700 African-American, Hispanic, and non-Hispanic white adolescents aged 12–17 were included in the final analyses. Of 199,700 youths, 148,360 youths who were eligible and responded to school-related items, were examined as supplementary analyses involving school-related factors. The final sample was evenly distributed across age (ages 12–14: 48.7%; ages 15–17: 51.3%) and gender (male: 50.8%; female: 49.2%). A majority of the youth identified as white (63.7%) while sizable proportions identified as Hispanic (20.5%) and African-American (15.8%). Roughly two in five (38%) reported an annual household income less than $40,000. 73.8% of the sample reported father’s presence in their households and over 98.8% were currently enrolled in school. A more detailed description of the NSDUH design is available elsewhere (SAMHSA 2014).

### Measures

*Drug Offers*. Each respondent was asked by a question “In the past 30 days, has anyone approached you to sell you an illegal drug?” Response options include 0 = No or 1 = Yes.

*Sociodemographic Factors*. The key sociodemographic characteristics include age, race/ethnicity (Black/African American, Hispanic, White), father in the house (yes, no), and school enrollment (yes, no). To account for the differential impacts of drug availability and use patterns by socioeconomic status (e.g. Humensky 2010) and region of residence (e.g., Gfroerer et al. 2007), annual family income ($0–$19,999, $20,000–$39,999, $40,000–$74,499, $75,000) and urbanity (Core Based Statistical Area, non-CBSA region) were included as control variables.

*Psychosocial Correlates.* As for psychosocial correlates, we examined individual-level (risk propensity and religiosity) and parent-related (parental affirmation and parental conflict) psychosocial factors. For Individual Factors, risk propensity was constructed based on two items (α = 0.73), asking “How often do you like to test yourself by doing something a little risky?” and “How often do you get a real kick out of doing things that are a little dangerous?” Dichotomized responses (never/seldom = 0, sometimes/always = 1) to the items were summed and treated as an ordinal variable (0 = low, 1 = medium, 2 = high) is consistent with prior studies (e.g., DeLisi et al. 2015; Salas-Wright et al. 2017b; Vaughn et al. 2016). Religiosity was examined using a 4-item scale (α = 0.72) reporting religious service attendance, private religious importance, importance and influence of religious beliefs, in consistent with prior studies (e.g., Farrington & Loeber 2000; Salas-Wright et al. 2014b).

In consistent to prior studies (e.g., Salas-Wright et al. 2017), we examined parental factors (i.e., parental affirmation and parental conflict) were constructed and examined. Parental affirmation was measured based on two items (α = 0.86), asking “During the past 12 months, how often did your parents let you know when you’ve done a good job?” and “During the past 12 months, how often did your parents tell you they were proud of you for something you had done?” Each response was dichotomized (never/seldom = 0, sometimes/always = 1), and then summed to be treated as an ordinal variable (0 = low, 1 = medium, 2 = high). Parental conflict was based on the following question: “During the past 12 months, how many times have you argued or had a fight with at least one of your parents?” The responses were coded as 0 = *0–2 times* and 1 = *3 or more times*.

*Behavioral Correlates.* As for behavioral correlates, we examined lifetime substance and other delinquent behaviors as well as past-year criminal justice involvement history. For Lifetime Substance Use, binary measures for lifetime alcohol, marijuana, and illicit drugs (hallucinogens, heroin, cocaine, inhalant, pain reliever, sedatives, stimulant, and tranquilizer) were used and the responses were coded 0 = *Never used* and 1 = *Used*. For other delinquent behaviors, self-reports on involvement of the following behaviors in the past year were examined: stealing something worth more than $50, a fight at school or work, and a group fight. Participant reporting involvement in respective behaviors was coded 1, and otherwise 0. For criminal justice involvement, a binary measure of self-reports on arrest/booking in 12 months (i.e., taken into custody and processed by the police or by someone connected with the courts) for breaking the law, not counting minor traffic violation, was used.

*School-Related Factors*. We examined academic engagement, grade, and school-skipping experience among participants who enrolled in school. In consistent with prior studies (e.g., Salas-Wright et al. 2014a), academic engagement were measured using 5 items (α = 0.77), including questions, such as, “How often felt school work meaningful?” and “How interesting are courses at school?” Grade was measured based on self-reports of average grade in the last/recent semester. Experience of school-skipping was measured based on the number of days participant skipped schools in the past month. The responses were coded as 0 = *Did not skip* and 1 = *Skipped once or more*.

### Statistical analysis

The statistical analyses were conducted in three steps. First, we examined the annual prevalence estimates of illicit drug offers from 2002 to 2014 for the full adolescent sample as well as for different subgroups by gender and race/ethnicity. Next, we tested the significance of the linear trends among the subgroups across sociodemographic characteristics, substance use pattern, and criminal justice involvement. Specifically, survey year was included as a continuous variable in the logistic regression models of illicit drug offers along with the sociodemographic characteristics as outlined by the Center for Disease Control and Prevention (2016). Lastly, we used logistic regression analyses to examine the associations between various sociodemographic, psychosocial, behavioral, and school-related correlates and illicit drug offers, controlling for sociodemographic characteristics. All estimates were weighted to account for the NSDUH’s stratified cluster sampling design according to the Substance Abuse and Mental Health Data Archive’s guideline (SAMHSA 2014).

## Results

### Trends in illicit drug offers among adolescents

**Fig. 1:**
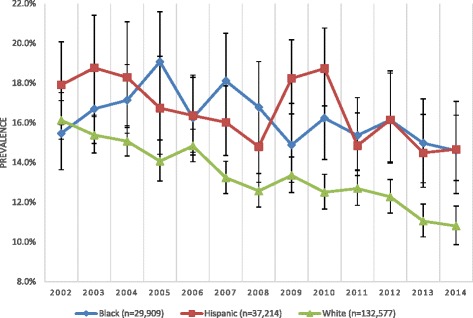
Trend in Past-month Drug Offers among Adolescents (aged 12–17): By Race/Ethnicity

Overall, there was a 24.5% decrease in drug offers overall, from 16.3% (15.6–17.0) in 2002 to 12.3% (11.5–13.2) in 2014 (AOR = 0.970, 95% CI = 0.965–0.975) (See Table 1). The decreasing trends were observed for both males and females, but the size of the reduction was much larger among males (29.5%) than females (16.5%) (not shown). When significance of the trends was tested among adolescents of different subgroups by sociodemographics (i.e., age, race/ethnicity, and urbanity), and behavioral health characteristics (lifetime alcohol, marijuana, and illicit drug use as well as past-12 months arrest/booking history), the reductions were found statistically significant except among life illicit drug users (AOR = 0.997, 95% CI = 0.989–1.004) (See Table 1). However, the magnitude of the decrease in drug offers among African-American and Hispanic adolescents was not as large as their white counterparts See (Fig. 1). While there was a 32.9% decrease (from 16.1% in 2002 to 10.8 in 2014) in drug offers among white adolescents, there were only 5.8 and 17.9% decreases among African-American and Hispanic adolescents, respectively.

**Table 1:** Test of Significance for Trends in Past-Month Drug Offers among Full-Sample and Subgroups by Sociodemographic/Substance Use/Criminal Justice Involvement: NSDUH 2002–2014

	Adolescents Aged 12–17 (*n* = 199,700)	
	AOR	(95% CI)
Full Sample	0.970^***^	0.965–0.975
Sociodemographic Subgroups		
*Age*		
Younger Adolescents (12–14)	0.969^***^	0.960–0.978
Older Adolescents (15–17)	0.971^***^	0.965–0.976
*Gender*		
Male	0.967^***^	0.961–0.973
Female	0.974^***^	0.967–0.981
*Race/Ethnicity*		
Black	0.981^**^	0.970–0.993
Hispanic	0.978^**^	0.967–0.990
White	0.963^***^	0.957–0.968
*Urbanity*		
Urban	0.971^***^	0.966–0.976
Rural	0.946^***^	0.929–0.964
Lifetime Substance Use		
*Alcohol*		
Never used	0.985^***^	0.977–0.993
Used	0.990^***^	0.984–0.996
*Marijuana*		
Never Used	0.972^***^	0.965–0.978
Used	0.981^***^	0.973–0.989
Other Illicit Drugs		
Never Used	0.976^***^	0.970–0.982
Used	0.997	0.989–1.004
Criminal Justice Involvement		
*Arrest/Booking History (in past 12 months)*		
No	0.975^***^	0.970–0.980
Yes	0.966^***^	0.948–0.983

Adjusted odds ratios adjusted for age, gender, race/ethnicity, household income, father in the house, school enrollment status, urbanity, and year. Significant odds ratios with a value of greater than 1.00 reflect an increase in trend and significant odds ratios with a value of less than 1.00 reflect a decrease in trend

*p < .05, **p < .01, ***p < .001

### Sociodemographic, psychosocial, behavioral, and school-related correlates of illicit drug offers

Table 2 displays the results from the logistic regression analyses on the link between sociodemographic/psychosocial/behavioral correlates and illicit drug offers. Youth reporting past-month drug offers were more likely to be African-American and Hispanic, to have household income less than $20,000 and $20,000–$39,999. Moreover, Youth reporting past-month drug offers were more likely to have medium or high risk propensity, have fought with parents three or more times in the past 12 months, ever drank alcohol or used marijuana or illicit drugs. Also, adolescents who ever stole more than $50 in the past 12 months, fought at school/work, were involved in a group fight, arrested/booked in the past 12 months, on probation or parole were more likely to be offered illicit drugs. On the other hand, adolescents reporting higher religiosity, and parental affirmation were less likely to be offered illicit drugs. Among those who responded to school-related factors (*n* = 148,360), lower grades and schooling-skipping were positively correlated with drug offers while higher academic engagement was negatively associated.

**Table 2:** Sociodemographic, Psychosocial, Behavioral, and School-related Correlates with Illicit Drug Offers among Adolescents: NSDUH 2002–2014

	Adolescents ages of 12–17 (*N* = 199,700)			
	Approached by someone selling illicit drugs in past 30 days?			
	No (%)	Yes (%)	AOR	95% CI
Sociodemographic Factors				
*Age*				
12–14	92.3 (92.1–92.5)	7.7 (7.5–7.9)	1.000	-
15–17	79.0 (87.6–79.3)	21.1 (20.7–21.4)	3.236^***^	3.134–3.343
*Gender*				
Male	83.1 (82.8–83.4)	16.9 (16.6–17.2)	1.000	-
Female	87.9 (87.6–88.2)	12.1 (11.8–12.4)	0.658^***^	0.633–0.684
*Race/Ethnicity*				
Black	83.7 (83.1–84.3)	16.3 (15.7–16.9)	1.140^***^	1.082–1.201
Hispanic	83.5 (83.0–84.0)	16.5 (16.0–17.0)	1.248^***^	1.191–1.308
White	86.6 (86.3–86.8)	13.5 (13.2–13.7)	1.000	-
*Household Income*				
Less than $20,000	83.9 (83.4–84.4)	16.1 (15.7–16.7)	1.078^*^	1.016–1.144
$20,000–$39,999	84.2 (83.8–84.7)	15.8 (15.3–16.2)	1.097^**^	1.037–1.160
$40,000–$74,999	85.9 (85.5–86.3)	14.1 (13.7–14.5)	1.020	0.972–1.071
$75,000 or higher	86.7 (86.3–87.1)	13.3 (12.9–13.7)	1.000	-
*Father in Household*				
Yes	86.5 (86.2–86.7)	13.5 (13.3–13.8)	0.803^***^	0.768–0.839
No	82.7 (82.2–83.1)	17.3 (16.9–17.8)	1.000	-
*Enrolled in School*				
Yes	85.7 (85.5–85.9)	14.3 (14.1–14.5)	1.000	-
No	69.1 (66.5–71.6)	30.9 (28.4–33.5)	1.550^***^	1.367–1.757
*Urbanity*				
Urban	85.2 (84.9–85.4)	14.9 (14.6–15.1)	1.000	-
Rural	90.1 (89.6–90.6)	9.9 (9.4–10.4)	0.628^***^	0.592–0.667
Psychosocial Correlates				
*Risk propensity*				
Low	91.3 (91.1–91.5)	8.7 (8.5–9.0)	1.000	-
Medium	83.3 (82.9–83.8)	16.7 (16.2–17.1)	1.987^***^	1.904–2.074
High	74.6 (74.1–75.0)	25.4 (25.0–25.9)	3.282^***^	3.157–3.411
*Religiosity*	-	-	0.447^***^	0.412–0.464
*Parental factors*				
Affirmation	-	-	0.690^***^	0.676–0.704
Fighting with parents				
No	89.4 (89.2–89.7)	10.6 (10.3–10.9)	1.000	-
Yes	82.2 (81.9–82.4)	17.9 (17.6–18.2)	1.951^***^	1.880–2.024
Behavioral Correlates				
*Lifetime Substance Use*				
Alcohol Use				
Never used	93.6 (93.4–93.8)	6.4 (6.2–6.6)	1.000	-
Used	72.3 (71.9–72.7)	27.7 (27.3–28.1)	4.630^***^	4.449–4.819
Marijuana Use				
Never used	91.3 (91.2–91.5)	8.7 (8.5–8.8)	1.000	-
Used	58.0 (57.3–58.7)	42.0 (41.3–42.7)	6.030^***^	5.813–6.256
Illicit Drug Use				
Never used	89.9 (89.7–90.1)	10.2 (9.9–10.4)	1.000	-
Used	65.1 (64.4–65.7)	34.9 (34.3–35.6)	4.301^***^	4.150–4.457
*Crime/Delinquency*				
Stole >$50				
No	86.8 (86.6–87.1)	13.2 (13.0–13.4)	1.000	-
Yes	52.6 (51.1–54.0)	47.4 (46.0–48.9)	4.873^***^	4.570–5.196
Fight at School/work				
No	88.6 (88.4–88.9)	11.4 (11.2–11.6)	1.000	-
Yes	73.4 (72.8–74.0)	26.6 (26.0–27.2)	2.989^***^	2.879–3.103
Involved in a Group Fight				
No	88.1 (87.9–88.3)	11.9 (11.7–12.1)	1.000	-
Yes	69.8 (69.1–70.6)	30.2 (29.4–30.9)	3.274^***^	3.154–3.398
Arrested/Booked (Past year)				
No	86.6 (86.4–86.8)	13.4 (13.2–13.6)	1.000	-
Yes	53.1 (51.5–54.7)	46.9 (45.4–48.5)	4.108^***^	3.846–4.389
On Probation				
No	86.1 (85.9–86.3)	13.9 (13.7–14.1)	1.000	-
Yes	53.3 (51.5–55.0)	46.7 (45.0–48.5)	3.761^***^	3.480–4.067
On Parole				
No	85.7 (85.5–85.9)	14.3 (14.1–14.5)	1.000	-
Yes	49.4 (45.3–53.6)	50.6 (46.4–54.7)	4.405^***^	3.667–5.293
School-related Factors (*n* = 148,360)				
Academic engagement	-	-	0.510^***^	0.492–0.528
Grades				
A	91.8 (91.5–92.2)	8.2 (7.8–8.5)	1.000	-
B	85.2 (84.9–85.6)	14.8 (14.4–15.1)	1.751^***^	1.663–1.844
C	78.2 (77.6–78.8)	21.8 (21.2–22.4)	2.602^***^	2.450–2.763
D	72.1 (70.8–73.3)	27.9 (26.7–29.2)	3.695^***^	3.393–4.024
Skipping School				
No	86.7 (86.4–87.0)	13.3 (13.0–13.6)	1.000	-
Yes	71.4 (70.5–72.2)	28.6 (27.8–29.5)	2.188^***^	2.078–2.303

Adjusted odds ratios adjusted for age, gender, race/ethnicity, household income, father in the house, school enrollment status, urbanity, and year

*p < .05, **p < .01, ***p < .001

## Discussion

Findings from the present study provide compelling evidence that fewer adolescents in the U.S. are being offered illicit drugs. The prevalence of being offered drugs among total adolescents dropped from 16.3% in 2002 to 12.3% in 2014, constituting a 24.5% reduction. Though not immediately comparable due to measurement differences, the prevalence was similar to the rates found in international studies such as Andreas and Pape (2015), where 17% of secondary school students in 2006 reported past-year cannabis offers in Norway. Encouragingly, the reduction was reported in different subgroups with behavioral health risks (i.e., substance use and criminal justice involvement history), as well as adolescents of different age, race/ethnicity, and urbanity characteristics. However, while we have observed a steady decline in adolescent drug use in recent years (Johnston et al. 2017), findings from the present study suggest that the prevalence of drug offers remained steady among the minority of youth who report using illicit drugs other than marijuana. Further investigation is recommended to see if illicit drug (other than marijuana) users are exposed to disproportionate risks of drug offers whereas non-users drive the decreasing trend in illicit drug use among adolescents.

Importantly, a closer inspection revealed persistent racial/ethnic disparities in drug offers. Although all racial/ethnic groups reported reduction in drug offers, the size of decreases among African-American and Hispanic was relatively marginal than their White counterparts. This racial/ethnic drug offer disparities led to the present situation in that significantly fewer white adolescents (10.8%) are offered drugs in 2014 than African-American (14.6%) and Hispanic (14.7%) adolescents unlike 2002 when there were no significant racial/ethnic differences were reported (African-American = 15.5%, Hispanic = 17.9%, and White = 16.1%). Given the crucial impact of drug offers on drug use initiation, later substance use behavior and associated disorders, further investigation is strongly suggested to understand recent drug offer patterns among African-American and Hispanic adolescents (Benjet et al. 2007; Wertz & Sayette 2001).

While more research is necessary to understand the mechanisms underlying declining drug offers, it is expected to be closely related to the recent decreases in substance use among youths. Given the substantial peer influence on drug offers and use among adolescents, the overall reduction in illicit drug use is likely to reduce the chances of getting offers via peer networks (Coombs et al. 1991; Neumark et al. 2012). Moreover, examining differences in peer dynamics and communicative strategies (e.g., the role of relational solidarity uniquely salient among Hispanic youth) across racial/ethnic subgroups may elucidate the different size of the drug offer reductions among racial/ethnic subgroups (Hecht et al. 1997; Moon et al. 1999). The observed differences in racial/ethnic drug offer trends support the need for culturally-grounded substance use prevention programs. For example, by incorporating culturally-sensitive prevention programs, such as the *keepin*’ *it* REAL (Hecht et al. 2003), that emphasize involvement of cultural competent helping professionals to better understand the target population, drug offers and use among different racial/ethnic subgroups may be interrupted.

In addition to the examination of the drug offer patterns, our findings suggested that a number of psychosocial risk behaviors as well as disadvantaged demographic characteristics were consistently associated with higher risk of drug offers among adolescents. Specifically, adolescents from non-white racial/ethnic groups and low-income families had higher risks of being offered drugs than their counterpart adolescents. We also found that adolescents reporting drug offers were more likely to have higher risk propensity, conflicts with their parents, to use substance, and to be involved in criminal justice system. For school-enrolled adolescents, those reporting drug offers were less likely to be engaged in academics, to receive higher grades, and to attend school more regularly.

Several limitations should be noted. First, all variables including socially undesirable behaviors, such as, substance use and criminal justice involvement were based on adolescents’ self-reports. This may have caused under-reporting and thus biased estimates. Second, data from the NSDUH are cross-sectional, thereby limiting any causal inferences. Despite these limitations, this study contributes to the adolescent health literature by presenting the recent trends of drug offers among adolescents with a wide-array of social and behavioral characteristics. Overall, findings suggest that adolescents are less likely to be offered drugs than a decade ago, but we also found persistent racial/ethnic disparities in these trends.
